# Gene Expression Analysis Platform (GEAP): A highly customizable, fast, versatile and ready-to-use microarray analysis platform

**DOI:** 10.1590/1678-4685-GMB-2021-0077

**Published:** 2021-12-17

**Authors:** Itamar José Guimarães Nunes, Mariana Recamonde-Mendoza, Bruno César Feltes

**Affiliations:** 1 Universidade Federal do Rio Grande do Sul, Instituto de Informática, Porto Alegre, RS, Brazil.; 2 Hospital de Clínicas de Porto Alegre (HCPA), Núcleo de Bioinformática, Porto Alegre, RS, Brazil.; 3 Universidade Federal do Rio Grande do Sul, Instituto de Biociências, Departamento de Genética, Porto Alegre, RS, Brazil.; 4 Universidade Federal do Rio Grande do Sul, Instituto de Biociências, Departamento de Biofísica, Porto Alegre, RS, Brazil.

**Keywords:** Microarray, R, gene expression, GUI, biomedical research

## Abstract

There are still numerous challenges to be overcome in microarray data analysis because advanced, state-of-the-art analyses are restricted to programming users. Here we present the Gene Expression Analysis Platform, a versatile, customizable, optimized, and portable software developed for microarray analysis. GEAP was developed in C*#* for the graphical user interface, data querying, storage, results filtering and dynamic plotting, and R for data processing, quality analysis, and differential expression. Through a new automated system that identifies microarray file formats, retrieves contents, detects file corruption, and solves dependencies, GEAP deals with datasets independently of platform. GEAP covers 32 statistical options, supports quality assessment, differential expression from single and dual-channel experiments, and gene ontology. Users can explore results by different plots and filtering options. Finally, the entire data can be saved and organized through storage features, optimized for memory and data retrieval, with faster performance than R. These features, along with other new options, are not yet present in any microarray analysis software. GEAP accomplishes data analysis in a faster, straightforward, and friendlier way than other similar software, while keeping the flexibility for sophisticated procedures. By developing optimizations, unique customizations and new features, GEAP is destined for both advanced and non-programming users.

## Introduction

Microarray analyses are applied to quantify the global gene expression from multiple biological samples in a specific condition (Blohm and Guiseppi-Elie 2001; Blalock 2003). Microarray data has successfully contributed to elucidate molecular mechanisms from complex diseases, such as Alzheimer disease (Itoh and Voskuhl 2017; Wang *et al.*, 2017a; Garranzo-Asensio *et al.*, 2018), Parkinson’s disease (Itoh and Voskuhl, 2017; Son *et al.*, 2017; Kong *et al.*, 2018) and multiple types of cancer (Vizkeleti *et al.*, 2017; Wang *et al.*, 2017b; Benkheil *et al.*, 2018; Duan *et al.*, 2018; Grindstad *et al.*, 2018; Li *et al.*, 2018; Yang *et al.*, 2018; Zhang *et al.*, 2018). This fast growth of biological datasets created a fertile ground for medical research that can be employed by other studies to further scientific knowledge (Marcotte and Date 2001; Zou *et al.*, 2015; Yin *et al.*, 2017). Hence, it is not a surprise that several publishers, such as Elsevier (see Elsevier in Internet Resources) and Nature (see Nature Scientific Data in Internet Resources) encourage or require authors to make their data availabel for the scientific community, whose files are publicly availabel in Gene Expression Omnibus (GEO) (see NCBI in Internet Resources) and The Cancer Genome Atlas (TCGA) (see National Cancer Institute in Internet Resources) for querying and validation.

Even nowadays, where the microarray technique is broadly availabel, the challenge of proper data analysis remains. In this sense, the three most known microarray manufactures, Affymetrix (see Affymetrix in Internet Resources), Agilent (see Agilent in Internet Resources), and Illumina (see Illumina in Internet Resources) offer specific software together with the analysis platform. However, these programs do not support file formats from other manufacturers and also may not provide a variety of options for statistical treatment, quality control, and results presentation, which imposes a significant barrier for researchers to obtain the best possible results.

An alternative is to create scripts using the R language (Ihaka and Gentleman, 1996; Crawley, 2012). There are a number of R packages provided by Bioconductor (Gentleman *et al.*, 2004) that include high-level functions for transcriptomic analysis, including *GEOquery* (Davis, 2007) for GEO data mining and *limma* (Smyth, 2005) for regression model fitting used in differential gene expression analysis. Additionally, there are R packages for microarray manufacturers as well, including *affy* for Affymetrix (Gautier *et al.*, 2004), *agilp* for Agilent (Chain, 2012), and *illuminaio*, *lumi* and *beadarray* for Illumina (Dunning *et al.*, 2007; Du *et al.*, 2008; Smith *et al.*, 2013). However, learning R can become troublesome and demand extra time for users without a programming background. Considering that dealing with algorithms is a less common expertise among biomedical researchers, a software dedicated for the regular, non-programming user can help accelerate scientific knowledge acquisition. In fact, creating accessible tools for non-bioinformatician users is already a topic of discussion (Kouskoumvekaki *et al.*, 2013).

Here we present the Gene Expression Analysis Platform (GEAP), a new, easy, flexible, customizable, and ready-to-use software that takes advantage of both Graphical User Interface (GUI) and R, to analyze microarray data from all platforms. GEAP was developed to translate the programming complexity underlying R’s microarray analyzes and optimize features that usually require manual editing or impose a slow learning curve. GEAP also brings several unique features for advanced and smoother analysis not seen in other similar software, such as automatic raw data retrieval from the GEO accession ID, followed by a robust file format checking, organized data storage, custom table building, custom filters, and multi-field columns. We tested GEAP in multiple types of datasets from different sources, backgrounds, platforms, and sample size to show its capabilities.

## Methods

### C# and GUI

GEAP was developed in two layers, consisting of a front-end GUI and a back-end R terminal (RTerm). The C*#*.NET programming language (Williams 2002) was chosen for GUI development since the .NET framework is majorly employed for Windows-based programs. The C*#* project was edited using Visual Studio 2017 environment, and the binaries were compiled in C*#* 7.3 using .NET Framework 4.6.1, which is compatible with Windows 7 to 10. Additionally, we developed a version for Ubuntu 18.04 using Mono 6.8.0 (see Mono Project in Internet Resources).

### Back-end R console and optimizations

Along with the GUI runtime, an RTerm is executed as a background process sharing memory and interacting with the GUI. To support the interconnection between the R environment and GUI, we developed an R package named *rgeap*, which comprises the entire set of functions being called from the front-end layer. This package was developed with the aid of RStudio (see RStudio Team in Internet Resources) running R version 4.0.3, and its primary purpose is to establish variables, methods, and package dependencies inside a single namespace to be accessed and called in RTerm at runtime. Thus, *rgeap* is not really a R package aimed to perform standalone differential expression analyses because it was developed as a technical solution. In this sense, some *rgeap* functions are responsible for serializing, receiving, and passing R objects to the GUI by RAM, thus avoiding slower data transfer rates through I/O. Moreover, this communication between the GUI and RTerm allowed GEAP to make use of the already published Bioconductor packages for microarray analysis. The main packages in the current implementation include: (i) *affy* (Gautier *et al.*, 2004) and *oligo* (Carvalho and Irizarry 2010) for reading and processing of Affymetrix CEL files; (ii) *makecdfenv* (Irizarry *et al.*, 2020) to handle custom CDF annotations for Affymetrix microarrays; (iii) *beadarray* (Dunning *et al.*, 2007) and *illuminaio* (Smith *et al.*, 2013) for Illumina IDAT and BGX files; (iv) *limma* (Smyth 2005) for differential expression analysis and statistical treatment of arrays, including those provided by Agilent and Illumina, and for linear model fitting; (v) *arrayQualityMetrics* (Kauffmann *et al.*, 2008) for array quality control; and (vi) *topGO* for gene ontology (GO) analyses (Alexa and Rahnenfuhrer 2010). In addition to the Bioconductor database, we used the *Rcpp* package to implement and optimize several R functions using the C++ language.

In some cases, the R functions were replaced by C*#* methods in the GUI for performance purposes. These features include:

- Download of multiple files using parallel Web requests: Since each Web request produce a connection delay, as occurs with single-threaded functions in R through the *GEOquery* package (Davis, 2007), multiple Web requests reduces download times when various files are requested. This was implemented using C*#* since R has no established support for multi-threading methods;
- Data table processing: When loading a tab-delimited file, R will scan the entire file before reading it. In our implementation, parallel computation is done to estimate the number of lines, followed by a lazy load of each line. In this second step, errors and forbidden entries (e.g., duplicates) are identified with the help of hash sets, thus reducing the complexity of some operations to *O(1)* instead of *O(n)*.
- Plotting and filtering: When result tables are produced in R, instead of depending on the R interpreter, the data is fully transferred to C*#* through memory. The transferred results data, now optimized, are used to generate dynamic plots and can be filtered faster through the pre-compiled methods.

### Availability and requirements

GEAP runs in Windows operating systems from 7 to 10, and in Ubuntu Linux from 18.04 onwards. In Windows, the Microsoft .NET Framework 4.6.1 or later is required to run the program and is installed by default in the latest Windows 10 updates. In Linux, a complete installation of Mono is required to run the executable files. Below, we listed GEAP’s minimum and recommended specifications. Although the program could run even below the minimum specifications, the amount of required space allocation in memory and processing makes any microarray analysis unfeasible in older computers. Similar to R, the more samples and probes included, the more hardware is expected.

### Software description

- Project name: GEAP
- Project home page: https://inf.ufrgs.br/geap
- Source code: https://github.com/nunesijg/rgeap
- Operating systems: Windows NT; Ubuntu
- Programming languages: C*#* and R
- License: MIT (GEAP); GNU LGPL (*rgeap*)
- Contact support: geapdevteam@gmail.com

*Minimum system requirements*

- RAM: 2 GB
- Processor: 2.4 GHz
- Operating system: Windows 7/8/10 or Ubuntu 18.04
- Free disk space: 1 GB
- Other requirements: .NET Framework 4.6.1 (Windows) or Mono 6.8.0 (Ubuntu)

*Recommended system requirements*

- RAM: 8 GB
- Processor: 2.4 GHz dual-core
- Operating system: Windows 7/8/10 or Ubuntu 18.04
- Free disk space: 10+ GB
- Other requirements: .NET Framework 4.6.1 (Windows) or Mono 6.8.0 (Ubuntu); and Network access for the options marked as “WEB”

## Results

### Application and GUI

**Figure 1 -:**
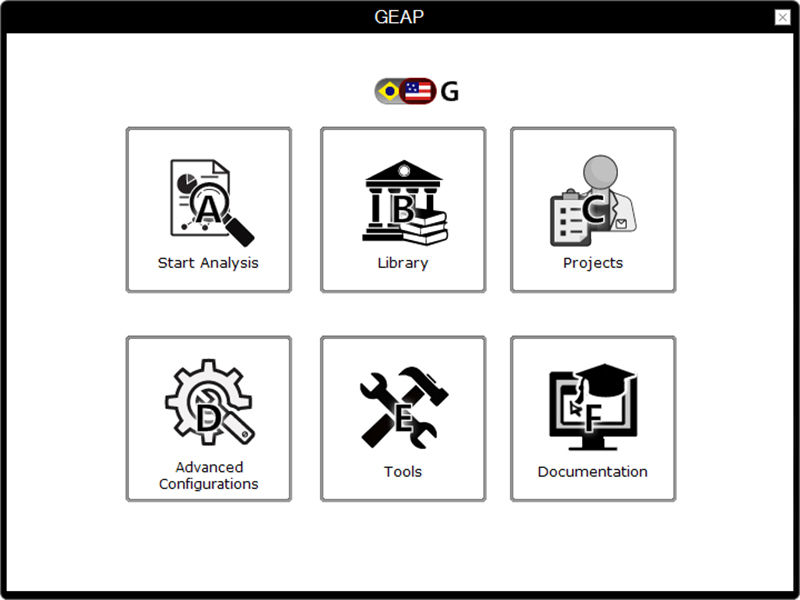
GUI Displayed in GEAP. Similar to a Website, GEAP divides each section into multiple pages, which can be accessed and returned by clicking the buttons. (A) Starts a new analysis, redirecting to pre-analysis menu; (B) Redirects to Library menu (see Figure 2 A-D); (C) Redirects to Projects menu (see Figure 2 E-J); (D) Displays the advanced options, including package updates, rendering quality and network connection; (E) Presents some additional tools, including a R console and the TypeChecker (see Figure S1); (F) Displays the program’s documentation; and (G) Toggles the displayed language.

In GEAP, the features, options, and GUI elements are displayed on individual pages, which can be accessed by clicking on labeled buttons (Figure 1). This page-by-page design was preferred, in contrast to a single workspace design (e.g., Microsoft Word or Adobe Photoshop), as an attempt to combine a clean and intuitive interface with the multiple machine states adopted by the program. This way, each section has its own set of interactive elements dedicated to a particular purpose, thereby preserving the complexity along with the analysis steps and preventing new users from becoming confused due to separate GUI elements.

### New features

GEAP takes an extra step from the primary R functions and provides new features created to expand the user’s possibilities to obtain, manage, customize, and analyze microarray data. We describe these features in each subsection below.

Automated dataset inspection: Every file used as input in GEAP is inspected and classified according to its extension and content. The format can be one of those listed in Table 1. During this inspection, the program checks the file format integrity, warning when data is corrupted or misformatted. This automatic feature spares the user from any need for prior dataset checking, manual editing, and from wasting time trying to process a corrupted dataset, which is a significant time-wasting process in microarray analyses.

**Table 1 -:** Data types supported by GEAP.

Full Name	Extension	Type	Manufacturer
Cell Intensity File	CEL (^*^.cel)	Sample	Affymetrix
Agilent Raw	Text (^*^.txt)	Sample	Agilent
Microarray Image	TIFF (^*^.tif)	Sample	Agilent; Illumina
GenePix Results	GPR (^*^.gpr)	Sample	GenePix
Bead Level Data	Text (^*^.txt)	Sample	Illumina
Pair Report	PAIR (^*^.pair)	Sample	NimbleGen
Markup RCC	RCC (^*^.rcc)	Sample	NanoString
Chip Definition File Package	R Package (^*^cdf.tar.gz); CDF (^*^.cdf)	Annotation	Bioconductor; Affymetrix
Probe Sequence Data	R Package (^*^probe.tar.gz)	Annotation	Bioconductor
Platform Design Info	R Package (pd.^*^.tar.gz)	Annotation	Bioconductor
Organism Database	R Package (^*^db.tar.gz)	Annotation; Gene Ontology	Bioconductor
GenePix Array List	GAL (^*^.gal)	Annotation	GenePix
Simple Omnibus Format in Text	SOFT (^*^.soft); Text (^*^.txt)	Sample; Series; Platform	GEO
Manifest File	BGX (^*^.bgx); Text (^*^.txt)	Annotation	Illumina
Tab-delimited file	TSV (^*^.tsv); Text (^*^.txt)	Sample; Series; Annotation	(User-provided)
Series Matrix	Text (^*^.txt)	Series	GEO
Intensity Data	IDAT (^*^.idat)	Series	Illumina
GO Annotation File	GAF (^*^.gaf)	Gene Ontology	Gene Ontology Consortium
	FB (^*^.fb)		
	MGI (^*^.mgi)		
	RGD (^*^.rgd)		
	TAIR (^*^.tair)		
	WB (^*^.wb)		
	ZFIN (^*^.zfin)		

**TypeChecker**: A separate GUI application, named TypeChecker, was developed to create new methods of file checking (Figure S1). This add-on presents visually related fields and variables for easy editing. Furthermore, the instructions for file checking are developed with blocks of visual commands, and not by command-line code. The only requirement of superficial programming occurs when statistical treatment of a specific file format is desired. This feature was implemented to spare the user from dealing with non-supported outputs from old or custom platforms. TypeChecker is capable of processing separate samples, platform data, and annotations.

**GEO metadata integration**: When processing GEO datasets, GEAP takes into consideration the file headers, which usually contain experimental descriptions of the data. In most cases, this metadata is found in one or more files in SOFT format, or in one SOFT format representing multiple GEO entries, where, in both cases, GEAP will read and merge their contents with the analyzable data. This metadata is used to improve access to experimental information along any analysis step, in contrast to depending on repeated queries in R. This is especially useful when GSM metadata is present, where GEAP integrates them into the individual samples, aiding their identification. This feature works with both single and dual-channel samples since GEAP separates the sample attributes according to the different channels.

**Figure 2 -:**
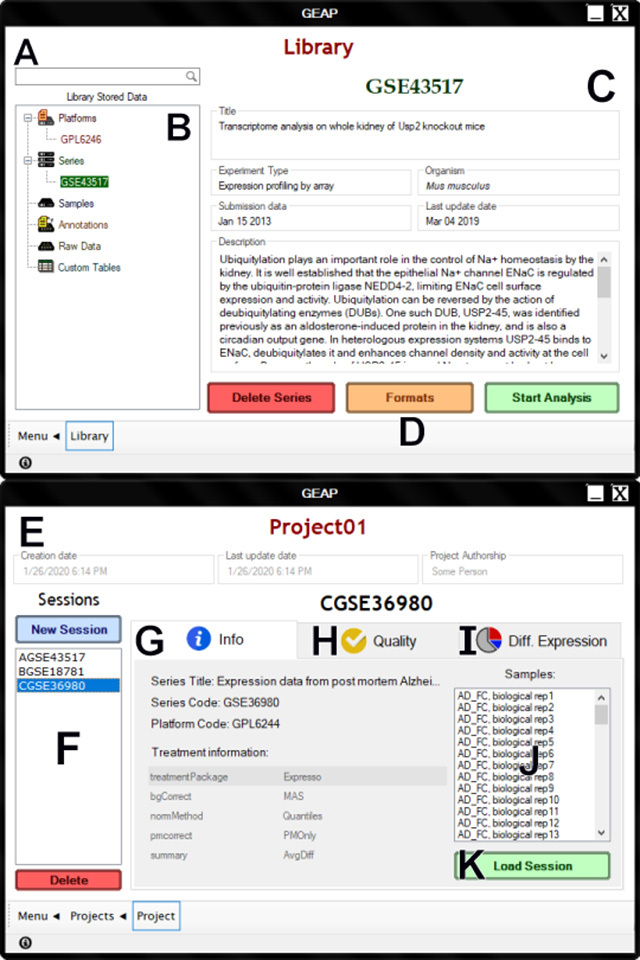
GUI for Library and Projects sections in GEAP. Users can use the Library to store microarray data and reload when needed, and use the Projects to save their own sessions from previous analyses. (A) Quick search bar for Library entries; (B) Currently stored Library data, categorized by Platforms, Series, Samples, Annotations, Raw data and Custom tables; (C) Information about the stored Library data; (D) Function buttons for the selected data. Series and samples can be stored in multiple formats since different statistical analyses can be applied on a single dataset, thus, generating distinct numeric matrices; (E) Project header; (F) List of saved sessions in the loaded project; (G) Session’s detailed info, appearing when a project session is selected; (H) Last quality analysis, if previously performed; (I) List of saved DE analyses, including the summary and preview plots from each previous DE result; (J) Availabel samples in the selected session; and (K) Button dedicated to reload the entire session. This spares the user the needless work of reanalyzing the entire array.

**Library**: In GEAP, users can save their input data, including GSE, GSM, GPL, custom tables, and annotation packages. Thereby, both metadata and content are indexed for further use into a portable database file that can be saved and loaded from another computer (Figure 2). All library data is serialized and stored inside a local SQLite database file for better compression and querying performance. According to benchmark tests, SQLite can provide approximately 35% of additional performance boost for binary data in comparison to reading the same data from File System (see SQLite in Internet Resources).

**Projects**: In contrast to other microarray analysis software, GEAP is capable of recording an entire analysis session, including the processed data and analysis results (Figure 2). This allows users to access the results section and interact again with plots and apply new filters, instead of repeating the entire analysis. This feature was designed to save time from retrieving and reanalyzing any previous information. Similar to “Library”, this feature allows to store the information offline into a portable database file that can be saved and loaded from another computer.

**Flexible input with custom tables**: The present methods for reading GSE, GSM, and GPL file formats require strictly formatted files as input. Although this covers most of the microarray series, there is so much heterogeneity among microarrays that unsupported formats may occur, such as those from platforms that are too old to possess a standardized file format. For these cases, GEAP offers the Custom Table section. In this section, any input file is accepted as long it is correctly formatted as a data table. Numeric columns can be treated as probe intensity values, while text columns can be used as probe attributes, except for the first column, which is the probe identifier. GEAP takes care of merging multiple tables with standard probe identifiers, which is useful when working with numerous tables with distinct probes. Furthermore, background signals and dual-channels can be selected and included as part of the preprocessing. Background correction and normalization methods from the *limma* package can be applied to numeric matrices derived from both single- and dual-channel arrays. In a typical microarray analysis, dealing with unformatted or heterogeneous inputs is hugely time-consuming. By using this feature, any table can be converted to a microarray dataset independently from the source platform or manufacturer, a task that has been usually only achieved through R programming.

**Filtering** Software from microarray manufacturers offer very basic filtering options to identify Differentially Expressed Genes (DEG) from comparison analyses, usually restricted to *log*_*2*_ FC and *p*-value. Programmatic filtering is possible in R, although it requires programming skills and may suffer performance drawbacks in large arrays. In contrast, GEAP allows users to customize the filtering of any table column and offers several options for numeric and character-wise matching. The *log*_*2*_ FC and *p*-value filters are presented by default, while other custom filters can be visually added and combined.

**Multi-field columns**: Numerous popular microarray platforms, in particular, those from Affymetrix, are known by probes identifying multiple genes. In GEO, the tables that represent such platforms concatenate genes into a single field, usually separated by ‘///’, making it difficult to dissociate the genes and work with them individually. In GEAP, these columns can be converted to a multi-field column, a data structure that indexes gene names into jagged arrays. This way, filters and analyses can be applied while taking into consideration the individual genes instead of the concatenated fields.

**Interactive scatter and volcano plots**: GEAP provide user-interactive scatter and volcano plots for visual exploration. Gene symbols and other attributes can be retrieved by hovering the mouse cursor over the plot points, and their selection is propagated to the data tables. Plot figures can also be saved to raster images, such as PNG, JPEG, BMP, TIFF, and GIF, and vectorial images, including SVG, WMF, and EMF. However, in contrast to the static plots generated in R, plots and charts in GEAP are optimized to dynamically change depending on the applied filters on the data tables, thereby increasing the interactivity and exploration capability over a large number of data points representing the filtered genes.

**Gene Ontology analysis**: GEAP supports Gene Ontology (GO) analyses to provide a biological meaning to gene expression results. The input genes are associated with biological processes and statistically evaluated by applying the *topGO* package (Alexa and Rahnenfuhrer, 2010). The relationship between genes and GO entries must be defined either from a chosen organism in a preset list or by providing a local GAF file. GEAP supports all organism database packages availabel in Bioconductor, currently comprising as much as 20 model organisms, and automatically solves package dependencies when necessary. For custom organism annotations, all the GAF and GAF-derived extensions listed in Table 1 are accepted and processed by GEAP. The accepted reference identifiers that establish the relationship between platform probe attributes and ontologies are: “Gene Symbol”, “Gene Name”, “Gene Alias”, “GenBank”, “Ensembl”, “Entrez”, “RefSeq”, “UniGene”, and “UniProt”. These fields are commonly present in both GPL data and organism databases.

**R source code generation**: GEAP keeps track of most actions along with the microarray data processing and analysis, mainly those executed with the aid of R environment, and aggregates them into an R script that can be accessed from an active analysis session. The generated code includes the user’s decision for method parameters and the input files and processed data, which can be exported in addition to the source code file. This way, experienced R programming users can also take advantage of GEAP’s interface to generate a reproducible script quickly and apply their methodology afterward. As the generated R scripts employ many functions developed in *rgeap*, this package is required to execute the code outside GEAP.

### Software validation

**Figure 3 -:**
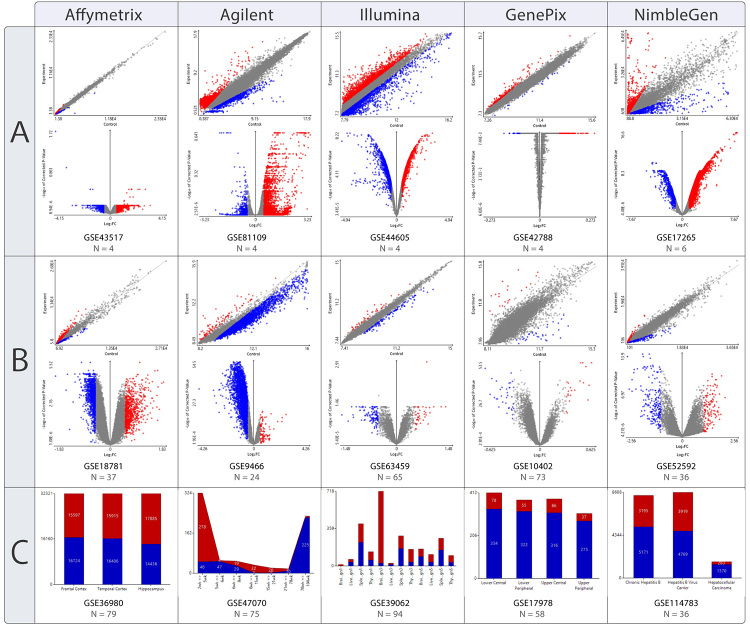
Plots for the results. Nine GSE were selected to validate GEAP’s capabilities for processing microarray data. Scatter plots use average expression values for experiment and control groups. Volcano plots use log_2_FC scale in X-axis and log_10_ of Corrected p-value in Y-axis. Bar and stack plots indicate the proportion between overexpressed (red) and underexpressed (blue) probes. (A) Scatter and volcano plots for comparisons between a small number of samples; (B) Scatter and volcano plots for comparisons between a large number of samples; (C) Bar plots for multiple comparisons (Affymetrix and Illumina) and stack plots for sequential comparisons (Agilent).

We had to make sure that this program can: (i) identify and process a reasonable variety of microarray data types; (ii) correctly deal with several amounts of samples, regardless of the chosen comparison mode; and (iii) efficiently display the results. We tested the program in the context of these three topics by analyzing 15 microarray datasets (Figure 3), choosing groups of three GSE from Affymetrix, Agilent, Illumina, GenePix, and NimbleGen. All GSE were directly loaded from Web mode, just by using the GSE code as input, satisfying the first topic. In each group, there was one GSE with a few samples, one with numerous samples divided between two phenotype groups and one with various samples divided into many phenotype groups. All datasets were analyzed from the raw format, and the default parameters of statistical treatment were applied using the R packages provided by the respective manufacturers.

Regarding the identification of microarray types, GEAP supports both single-channel and dual-channel microarrays. The program can distinguish when one or two-colors are used during pre-analysis, depending on the dataset file contents. The main difference can be observed when determining the samples for differential expression comparison. Samples are typically distributed to different groups for single-channel arrays. However, for dual-channels, once a sample is assigned, for example, to the control group, GEAP automatically assigns the same sample in the experimental group. Users can visually distinguish the channels when two-color microarrays are employed by the colors of the circles that precede the sample names. When sample information from GEO is availabel, the circles are painted with the colors emitted by the labels (e.g., red for Cy5, green for Cy3, yellow for biotin, and blue for some Alexa products). If this information is not availabel, GEAP deduces the channel color based on the file contents. Three of the chosen GSE to validate the software were dual-channel (GSE9466, GSE10402, and GSE 17978), which GEAP successfully identified.

After pre-processing, the samples were distributed for differential expression comparisons, with the comparison results being plotted (Figure 3). These plots indicate that GEAP can deal with many samples and comparisons, being limited only by the local RAM. The generated plots also presented some expected results, such as higher dispersion and flattened volcano plots (i.e., less statistical significance) in groups with fewer samples. Few samples, in general, make noise more apparent between the array intensities, while a higher number of samples usually yield a more precise and realistic distribution of probe intensities.

Even in the multiple group’s comparisons, a biased result might be observed for the Illumina platform, where the proportion of underexpressed genes may be imprecise due to the low sample amount (Figure 3). On the other hand, the sequential comparison for Agilent was performed using many samples, and the plot depicts the lifelong gene expression changes in a *Rattus norvegicus* kidney. These observations fulfill the second topic since GEAP presents different modes to analyze data and present adequate results regardless of the chosen comparison.

**Figure 4 -:**
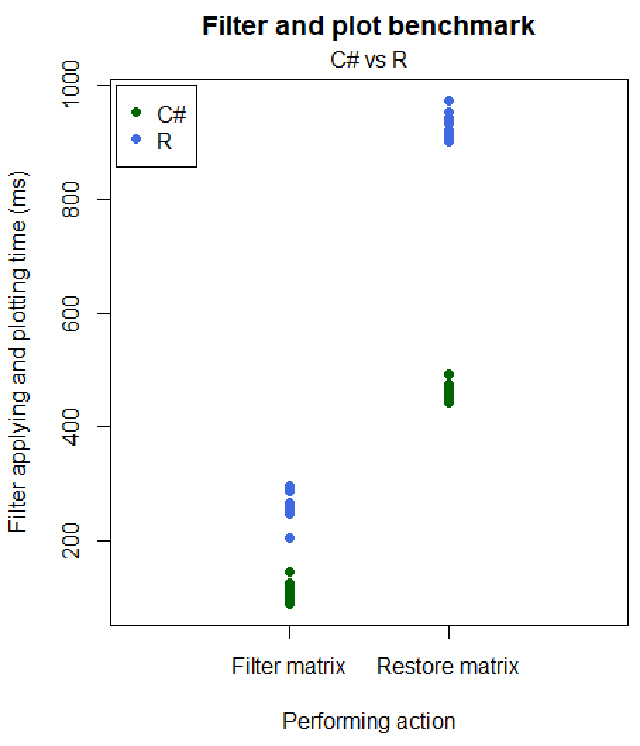
Filtering and plotting performance. When comparison results are produced, GEAP uses his data frame structure compiled in C*#* instead of depending on the R environment. As a result, GEAP finishes the same procedures with almost twice the performance. These observations were based on 48 benchmark tests performed separately in GEAP and the R console. We restricted the time range from before the matrix redefinition to the moment after matrix plotting. In the left column, the measure refers to when the matrix is filtered, and the obtained values are plotted, while in the right column, the matrix was returned to its unfiltered state.

Finally, we tested the efficiency of displaying results in GEAP. While the user can interact with the plots in real-time, there is a processing time to generate the plots every time a new filter is applied. Although this limitation is real for R and GEAP, we observed, after 96 benchmarking tests (48 for C*#* and 48 for R), that GEAP had a significant performance improvement to generate plots (Figure 4) over R. These benchmarks evidenced that GEAP not only plots faster, but the plots also are user-interactive, in contrast to the static R plots.

## Discussion

### Using GEAP for microarray data analysis

**Figure 5 -:**
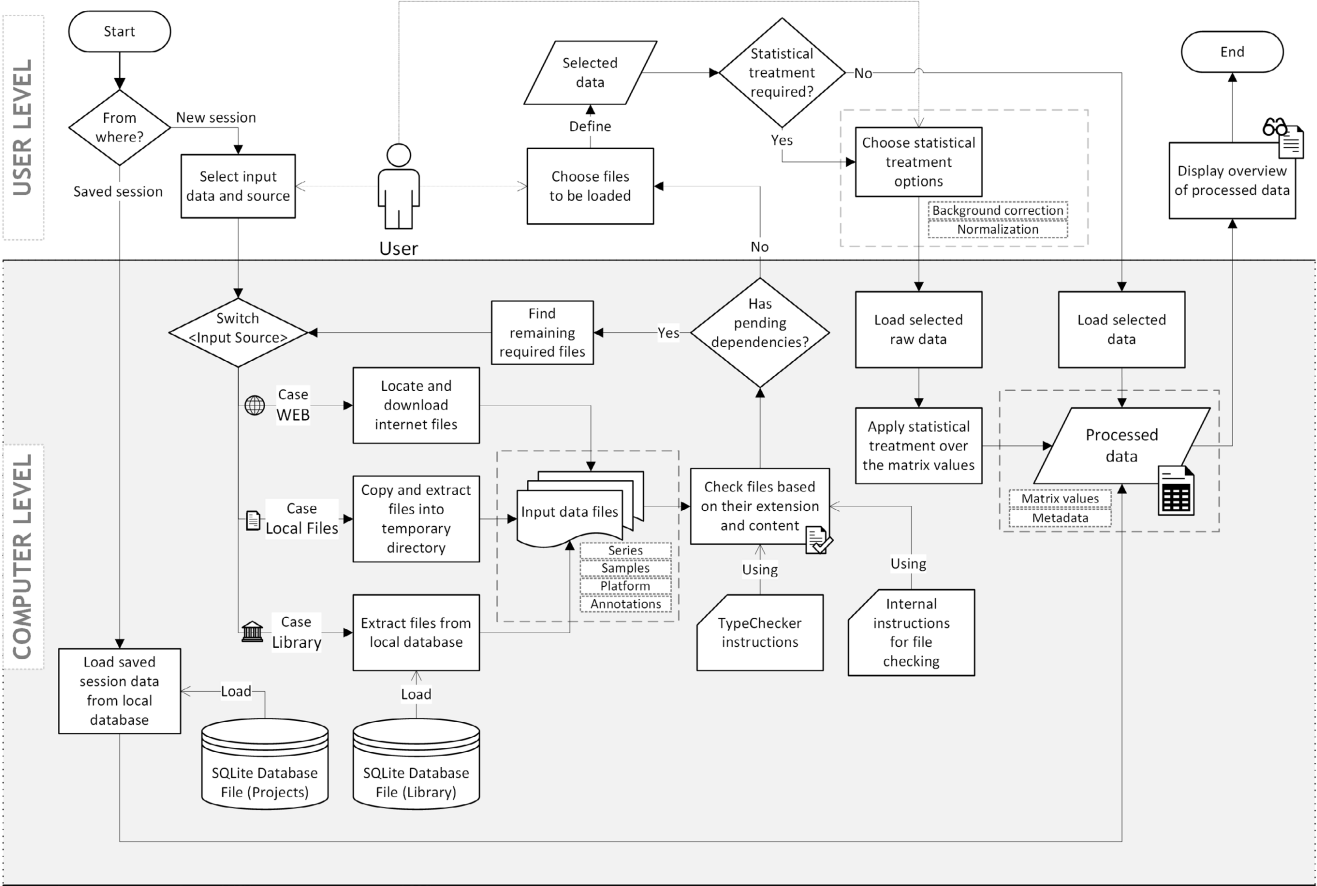
Pre-analysis flow diagram. The User Level represents the front-end layer where users interact with the program’s features from pages and windows forms. The Computer Level represents the internal logic to be executed depending on the user’s choice. Diamonds represent a condition based on the current program’s state, where switches select one of the multiple cases based on the previous action; rectangles represent either an interface operation at the User Level or an internal procedure at the Computer Level, where the user’s decision points are indicated with dashed arrows; rectangles with a cut-off corner represent included features that participate in the step; and cylinders, parallelograms, and wavy rectangles represent data as specified in their labels.

**Figure 6 -:**
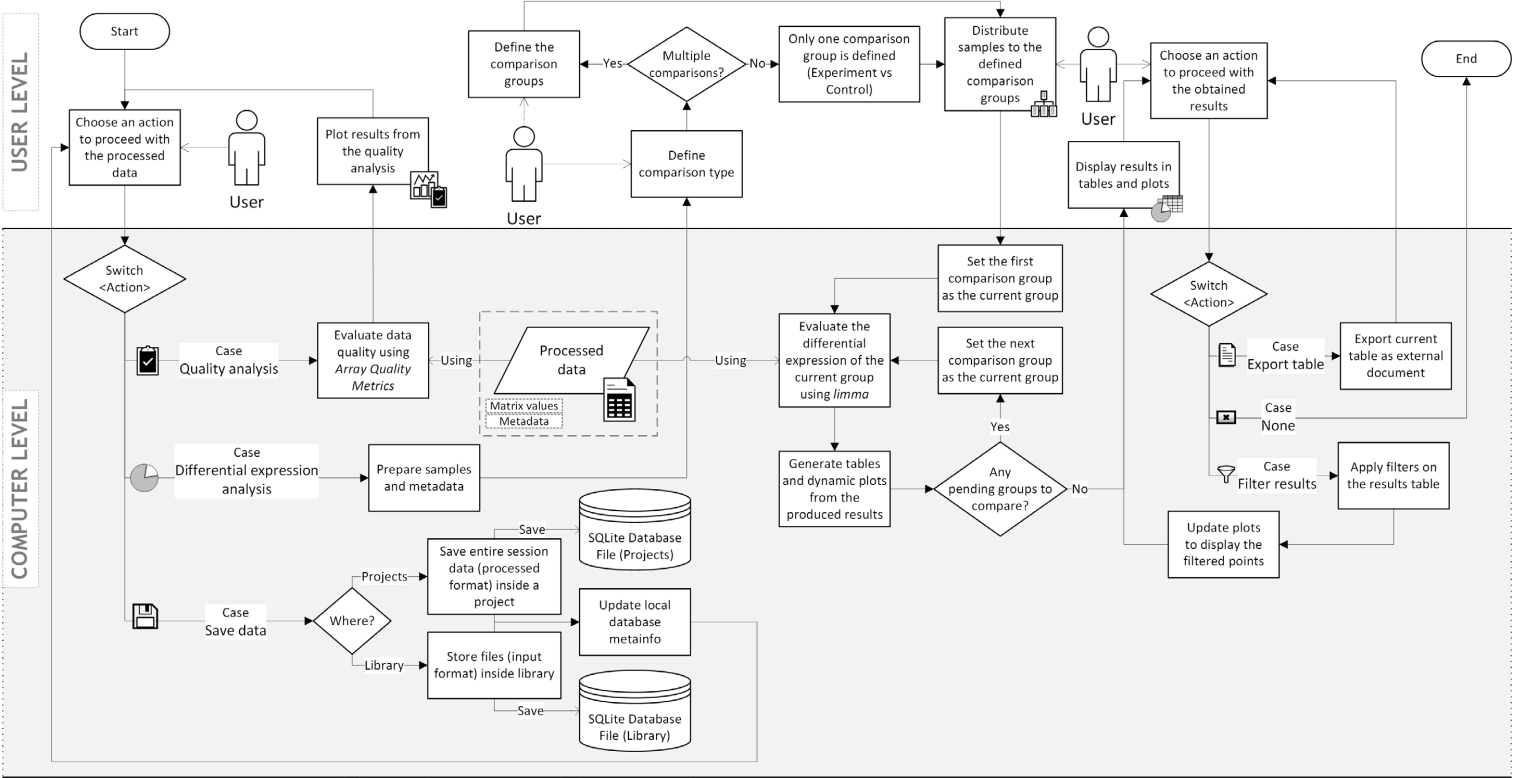
Analysis flow diagram. The User Level represents the front-end layer where users interact with the program’s features from pages and windows forms. The Computer Level represents the internal logic to be executed depending on the user’s choice. Diamonds represent a condition based on the current program’s state, where switches select one of the multiple cases based on the previous action; rectangles represent either an interface operation at the User Level or an internal procedure at the Computer Level, where the user’s decision points are indicated with dashed arrows; and cylinders and parallelograms represent data as specified in their labels. The “using” arrows indicate the inclusion of the specified data in that step.

The complete analysis is composed by multiple steps, starting from the data processing and analytical treatment (i.e., pre-analysis, see Figure 5) for quality evaluation and differential expression (in other words, the main analysis, see Figure 6). We describe, in each subsection below, the detailed procedures when analyzing microarrays using GEAP.

**Pre-analysis using GSE or GSM as input**: Before pre-analysis, transcriptomic data sets must be localized within the GEO database and downloaded. In this sense, a few definitions are required: (i) a group of samples related to a study is denominated GEO series (GSE); (ii) probe values related to a GEO sample are designated GSM; and (iii) probe identifiers associated with a specific GEO platform, named GPL.

In GEAP, from the initial page, following “Start Analysis”, three options of input data are presented: “Series”, “Separated Samples” and “Custom Table”. The first and second options share the same parameters, except that the first accepts a single GSE file, and the later accepts multiple GSM files. When working with GSE or GSM, users must choose if the arrays will be in the previously treated format (i.e., SOFT format) or in RAW form, as well as the method to obtain the GPL corresponding to the selected series or samples. All data concerning GSE, GSM, and GPL, including dependency files, can be obtained by three methods: Web, Local File, and Library. Both “Local File” and “Library” methods use data exclusively from local secondary storage. If “Web” option is chosen, the program will send a request to the GEO database to verify if the inserted GSE or GSM codes exist, and then checks data integrity. This happens for both SOFT or RAW data. Occasionally, a single GSE may associate with multiple GPLs. In these cases, GEAP gives the choice of which platform will be loaded and automates the process of separating platform-specific files.

After checking all input files, GEAP prompts a dialog listing these files, allowing users to select which files will be taken into account. This is useful, for example, when an entire GSE with multiple sample files was downloaded, but only a subset of samples is desired. Moreover, if data was provided in RAW format, the user is prompted with the availabel preprocessing methods for this format. The preprocessing methods correspond to functions and packages used in R for microarray data treatment, including background correction and normalization. Currently, there is a total of 32 statistical methods that users can choose. GEAP readily distinguishes single and dual-channel experiments.

**Overview and quality control**: At the end of pre-analysis, the GUI redirects the user to a section displaying the entire overview of the obtained microarray (Figure S2) that can be saved into the GEAP library. There is also an option to export the processed data as text file (TXT and SOFT formats) or R session (RData and RDS formats), which can be reloaded in R. The next steps are depicted in Figure 6.

Before comparing samples, it is worth accessing the overall array quality control. Although statistical treatment helps to correct moderate amounts of noise, it does not change the overall array quality nor repair poorly performed experimental procedures. In this sense, the consistency and scale of numeric values between samples and probes may be tested and plotted for more in-depth validation to detect incoherent expression profiles and outliers. If there are samples outliers that do not correspond to the context of the study, they can be removed from further analyses. In this sense, GEAP applies *arrayQualityMetrics* function from the homonym package. The generated plots describing each section are separated between the section tabs, as depicted in Figure S3. If outliers are identified in one or more sections, the section number is presented in the quality results table (Figure S3); otherwise, an “OK” message is displayed.

In the end of the quality analysis, a report describing the results becomes availabel, as well as the output plots, which can be accessed by the “Full Report” button. Users can opt to export all results as an HTML document, similar to the *arrayQualityMetrics* report by clicking on the “Export Results” button.

**Differential expression analysis**: In this step, the samples are presented as group items on a list box, which are distributed between other list boxes representing the experimental and control groups, as shown in Figure S4. GEAP provides two additional modes, aside from the regular control *versus* experimental option. The first is multiple group comparison and sequential comparison, where two or more experiment-versus-control pairs are created. The second is the temporal comparison, where the samples are distributed in separate groups named “steps”, and the comparisons are made taking “steps” as the control group and the next “steps” as the experiment group. In addition, these “steps” can be compared between distinct layers of experimental conditions. For example, it is possible to combine different treatments with time-dependent tests in one single analysis.

If a GPL (from GSE and GSM) or attribute values (from custom tables) are obtained during pre-analysis, the user can select which probe attributes will appear in results. The “Gene Symbol” option, as well as any other gene identifiers present in the GPL, is checked by default. For *p*-value correction, the availabel methods are FDR (Benjamini and Hochberg, 1995), Benjamini-Yekutieli (Benjamini and Yekutieli, 2001), Bonferroni, Hochberg (Hochberg, 1988), Holm (Holm, 1979) and Hommel (Hommel, 1988).

**Results presentation and management**: Finally, a section containing the general results overview is displayed (Figure S5), with the DEGs being depicted in a pie chart. If multiple comparison mode was selected, a bar chart is displayed instead.

**Figure 7 -:**
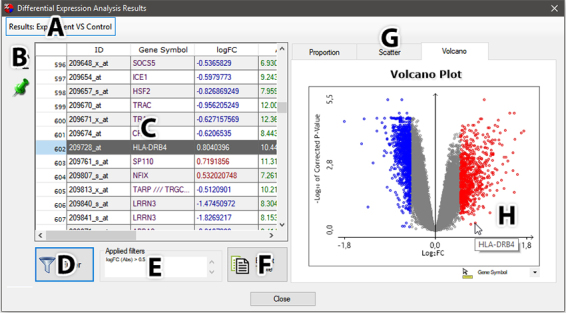
Full table of comparison results. (A) Comparison result selector. If multiple comparisons were performed, various items are displayed; (B) column sorting button and ``Pin first column’’ button; (C) the complete results table; (D) filtering functions; (E) currently applied filters; (F) table exporting; (G) availabel plots, separated between tabs; and (H) plot dynamic interacting with the mouse. If filtering was applied, only the filtered (blue and red) points become interactive, and the selection propagates to the full table.

If the sequential comparison mode was selected, a stacked area chart is displayed to illustrate the sequential change of proportions between DEGs. Through the “Full Table” button, a separate window is displayed with one half of its area occupied by a results table and another half by the dynamic plots (Figure 7). In this specific case, a pop-up window was favored to allow scale adjusting. The table is paged to support the exploration of extensive amounts of data. The plots are interactive and react to mouse hover, click, and area selection, whereas every node selection reflects into the table.

The initial output is not filtered by default, unless if previously specified in “Advanced Options”, hence, no clear threshold can be observed between DEG. By clicking on “Filter”, the filter dialog box is shown, presenting a couple of default filtering options for logFC and *p*-value. Below these options, a grid is availabel, where any textual or numeric column can be used as a filtering parameter. Setting filters will affect all comparison results on the entire analysis. In the table, only the rows matching the filter will be displayed, while in the plots, the nodes corresponding to non-filtered values become obscured and unselectable. Finally, after applying the desired filters, users can export the filtered tables and plots in a ready-to-publish format.

### Comparison to similar software: How is GEAP different?

In this section, we will describe GEAP’s differences from software currently used for similar purposes. An overview of the comparisons between specific features from each program is presented in Table 2. 

**Table 2 -:** Comparison between GEAP and other microarray software with similar features. Aside from the comparisons made below, GEAP is the only software that contains a library storage, project section, download and file integrity check directly from GEO, thus we did not include a column for those features.

Sofware	Functional requirements	Runtime environment	Supported sources and manufacturers	Internal data storage	Input and dependency handling	Latest version and release date
GEAP	None (Windows); Mono (Linux)	Standalone	Agilent; Affymetrix; Illumina; GenePix; NimbleGen; NanoString; GEO; User (Custom tables)	Files (Library); Sections (Projects); Results	Automatic and Manual	0.4.0 March 2021
Transcriptome Analysis Console	None	Standalone	Affymetrix	None	Manual (Except annotations)	4.0.2 January 2019
GenomeStudio	None	Standalone	Illumina	None	Manual	2011.1 June 2011
Genomic Workbench	None	Standalone	Agilent	None	Manual	7.0 December 2015
GEO2R	Network connection	Web	GEO (curated only)	None	Automatic only	(Not versioned) April 2021
Babelomics	Network connection	Web	Agilent; Affymetrix; Illumina; GEO	Uploaded Files	Manual (Except annotations)	5 July 2018
Chipster	Java; Virtual Machine (v3.x); Web (v4, requires institutional account)	Virtual Machine (v3.x); Web (v4)	Agilent; Affymetrix; Illumina; GEO	Files (inside Virtual Machine); Server (v4)	Automatic and Manual	v4 December 2020
eUTOPIA	R	R	Agilent; Affymetrix; Illumina; GEO	None	Automatic and Manual	(Not versioned) September 2019
ShinyGEO	Network connection	Web	GEO	None	Automatic only	(Not versioned) April 2021

We highlight that some implementations are exclusive to GEAP and not featured in other programs, including: (i) downloading the entire array in raw format and solving its dependencies by only providing a GEO accession ID; (ii) GEO metadata integration to analysis workspace; (iii) support for custom tables; (iv) project management; (v) library for file storage; and (vi) fully customized filtering of results. TypeChecker add-on also separates GEAP from other software because it allows the user to expand the program’s support to more file formats.

Affymetrix’s Transcriptome Analysis Console (TAC) features data pre-processing, statistical treatments, multiple comparisons, and interactive plots, just as GEAP, but only accepts Affymetrix microarrays. TAC provides more plots such as HeatMaps and Chromosome views, while GEAP supports customizable tool-tips linked to the data points, making GEAP plots easier to explore. Illumina’s GenomeStudio and Agilent Genomic Workbench provides features related to single-nucleotide polymorphisms, which is not implemented in the current GEAP version; however, both of them only accept their platform-specific files.

Some platform-independent programs can also be compared with GEAP. Babelomics (Alonso *et al.*, 2015; see Babelomics in Internet Resources), for instance, can process Affymetrix, Agilent, and GenePix microarrays, although it currently has no Illumina data support. Illumina datasets are the most heterogeneous in terms of data structure and file extension, which automatically impose an extra challenge for data handling. As an online application, Babelomics also requires uploading the files to be processed, where GEAP can retrieve data from GEO, or access local data. ShinyGEO, in contrast, does not provide a data storage service and the input is automatically imported by providing the accession ID (Dumas *et al.*, 2016), although this feature is limited to the author’s preprocessed format and has no support for RAW data, not offering the same raw data processing and dependency-solving that GEAP provides. Moreover, Chipster (Kallio *et al.*, 2011) is an example of an offline application that supports data from GEO and provides tools for statistical treatment, quality analyses, filtering, and results plotting. Chipster also is capable of importing data directly from GEO, although similarly to ShinyGEO, this feature is restricted to the author’s preprocessed format from GEO. Furthermore, Chipster is a server-based program and is expected to be installed inside a virtual machine to run at maximum performance. GEAP, in contrast, is portable, and downloading the application is sufficient to run with its full potential, being more user-friendly for non-programming users.

A more recent example is eUTOPIA (Marwah *et al.*, 2019), a GUI tool developed in R that makes use of different Bioconductor packages. Some additional plots that are provided by eUTOPIA are not currently present in GEAP. However, eUTOPIA depends on the installation of R and many dependencies to run, thus not relatively ready-to-use if compared to GEAP, which does not demand any contact between the user and R environment during the analyses.

In a final note we want to highlight that GEAP was developed with portability in mind by avoiding installation and software requirements (except Mono in the Ubuntu version); hence it can work from a USB flash disk in any Windows machine. It also extends this portability by including local storage for the analyzed data in RAW and processed formats. Furthermore, it was developed to maximize the automation of microarray analysis by developing a processing method for every RAW data format and encapsulating the procedures for quality analysis, differential expression comparisons, and Gene Ontologies in a user interface to avoid manual data handling or programming knowledge. This automation was extended to the point that an entire dataset in RAW format and its dependencies could be loaded (locally or from the internet) and fully processed into a common analysis workflow by providing a GEO accession identifier (GSE, GDS, or multiple GSM) without the user’s interference. There is a set of processing options for every use case, which is also an exclusive feature to GEAP. Another crucial mindset that was embedded into GEAP is the possibility to overcome possible analytical road-blocks with TypeChecker, which allows the user to process samples, platform data, and annotations in an easy and custom way. This combination of capabilities related to automation, versatility, and portability is what makes GEAP unique from other microarray software, even though it may share similarities in particular points.

## Conclusions

The majority of the scientific community that employs microarray analysis are molecular biologists and biomedical researchers with little or no programming background, making it harder for them to have the best pipeline availabel to analyze microarray data. Although other existing software provides state-of-the-art microarray analysis for the non-programming user, GEAP still offers the most significant flexibility and customization options of them all. To ensure the friendliest interface, GEAP takes extra steps in all layers of a microarray pipeline from data retrieval, project and library organization, to a myriad of statistical options, table, and data customization, to faster filtering and results plotting. GEAP is also availabel in two languages: Portuguese and English. A Japanese version of this software is also being considered for future releases.

Currently, GEAP offers only single and dual-channel microarray data analysis, but we are currently studying a possibility to automate RNA-seq analysis in GEAP in the future. This is a hard task, because the RNA-seq pipeline is massively customized from preprocessing to analysis, and changes according to numerous variables, not being nearly as straightforward as microarrays. Likewise, the massive demand for RAM makes it difficult to optimize the pipeline with a GUI application on a regular computer (*e.g.*, GALAXY, for example, runs on a large server). But advances on preprocessing steps of RNA-seq can be expected soon. Other add-ons are already being developed for GEAP, including plug-in development tools and plug-in support, aiming to expand the software’s capabilities. Such plug-in system is planned to allow users to create pipelines, macros, and R package integrations through instruction blocks similarly to TypeChecker, as an effort to further increase GEAP’s support to other large-scale analyses. Besides, since GEAP was initially intended to run in Windows only, the Ubuntu version of GEAP is still in its early implementation stages. It has limited functionality compared to the Windows version, and more development is in the process to compensate these limitations for the next releases.

## Supplementary Material

The following online material is availabel for this article:

Figure S1 - TypeChecker’s main workspace with a use case example.

Figure S2 - Overview section that summarizes the information extracted from pre-analysis.

Figure S3 - Quality results section.

Figure S4 - Distribution of samples between experimental and control groups in a study case involving tumors.

Figure S5 - Summary of diﬀerential expression analysis results obtained by comparing the groups indicated in Figure S4.

## References

[B1] Alexa A, Rahnenfuhrer J (2010). topGO: enrichment analysis for gene ontology. Bioconductor. R package version 2.42.0.

[B2] Alonso R, Salavert F, Garcia-Garcia F, Carbonell-Caballero J, Bleda M, Garcia-Alonso L, Sanchis-Juan A, Perez-Gil D, Marin-Garcia P, Sanchez R (2015). Babelomics 5.0: Functional interpretation for new generations of
genomic data. Nucleic Acids Res.

[B3] Benjamini Y, Hochberg Y (1995). Controling the false discovery rate: A practical and powerful
approach to multiple testing. J R Stat Soc Ser B.

[B4] Benjamini Y, Yekutieli D (2001). The control of the false discovery rate in multiple testing under
dependency. Ann Stat.

[B5] Benkheil M, Paeshuyse J, Neyts J, Van Haele M, Roskams T, Liekens S (2018). HCV-induced EGFR-ERK signaling promotes a pro-inflammatory and
pro-angiogenic signature contributing to liver cancer
pathogenesis. Biochem Pharmacol.

[B6] Blalock EM (2003). A Beginner’s Guide to Microarrays.

[B7] Blohm DH, Guiseppi-Elie A (2001). New developments in microarray technology. Curr Opin Biotechnol.

[B8] Carvalho BS, Irizarry RA (2010). A framework for oligonucleotide microarray
preprocessing. Bioinformatics.

[B9] Chain B (2012). agilp: Agilent expression array processing
package. R package version 3.19.0.

[B10] Crawley MJ (2012). The R book.

[B11] Davis S, Meltzer P (2007). GEOquery: a bridge between the Gene Expression Omnibus (GEO) and
BioConductor. Bioinformatics.

[B12] Du P, Kibbe WA, Lin SM (2008). lumi: A pipeline for processing Illumina
microarray. Bioinformatics.

[B13] Duan S, Gong B, Wang P, Huang H, Luo L, Liu F (2018). Novel prognostic biomarkers of gastric cancer based on gene
expression microarray: COL12A1, GSTA3, FGA and FGG. Mol Med Rep.

[B14] Dumas J, Gargano MA, Dancik GM (2016). shinyGEO: A web-based application for analyzing gene expression
omnibus datasets. Bioinformatics.

[B15] Dunning MJ, Smith ML, Ritchie ME, Tavaré S (2007). beadarray: R classes and methods for Illumina bead-based
data. Bioinformatics.

[B16] Garranzo-Asensio M, San Segundo-Acosta P, Martínez-Useros J, Montero-Calle A, Fernández-Aceñero MJ, Häggmark-Månberg A, Pelaez-Garcia A, Villalba M, Rabano A, Nilsson P (2018). Identification of prefrontal cortex protein alterations in
Alzheimer’s disease. Oncotarget.

[B17] Gautier L, Cope L, Bolstad BM, Irizarry RA (2004). Affy - Analysis of Affymetrix GeneChip data at the probe
level. Bioinformatics.

[B18] Gentleman RC, Carey VJ, Bates DM, Bolstad B, Dettling M, Dudoit S, Ellis B, Gautier L, Ge Y, Gentry J (2004). Bioconductor: Open software development for computational biology
and bioinformatics. Genome Biol.

[B19] Grindstad T, Richardsen E, Andersen S, Skjefstad K, Donnem T, Ness N, Nordby Y, Bremnes RM, Al-Saad S, Busund L-T (2018). Progesterone receptors in prostate cancer: Progesterone receptor
B is the isoform associated with disease progression. Sci Rep.

[B20] Hochberg Y (1988). A sharper Bonferroni procedure for multiple tests of
significance. Biometrika.

[B21] Holm S (1979). A simple sequentially rejective multiple test
procedure. Scand J Stat.

[B22] Hommel G (1988). A stagewise rejective multiple test procedure based on a modified
Bonferroni test. Biometrika.

[B23] Ihaka R, Gentleman R (1996). R: A language for data analysis and graphics. J Comput Graph Stat.

[B24] Irizarry RA, Gautier L, Huber W, Bolstad B (2020). makecdfenv: CDF Environment Maker. R package version 1.66.0.

[B25] Itoh Y, Voskuhl RR (2017). Cell specificity dictates similarities in gene expression in
multiple sclerosis, Parkinson’s disease, and Alzheimer’s
disease. PLoS One.

[B26] Kallio MA, Tuimala JT, Hupponen T, Klemelä P, Gentile M, Scheinin I, Koski M, Käki J, Korpelainen EI (2011). Chipster: User-friendly analysis software for microarray and
other high-throughput data. BMC Genomics.

[B27] Kauffmann A, Gentleman R, Huber W (2008). arrayQualityMetrics-- A bioconductor package for quality
assessment of microarray data. Bioinformatics.

[B28] Kong P, Lei P, Zhang S, Li D, Zhao J, Zhang B (2018). Integrated microarray analysis provided a new insight of the
pathogenesis of Parkinson’s disease. Neurosci Lett.

[B29] Kouskoumvekaki I, Shublaq N, Brunak S (2013). Facilitating the use of large-scale biological data and tools in
the era of translational bioinformatics. Br Bioinform.

[B30] Li SY, Wu HC, Mai HF, Zhen JX, Li GS, Chen SJ (2018). Microarray-based analysis of whole-genome DNA methylation
profiling in early detection of breast cancer. J Cell Biochem.

[B31] Marcotte EM, Date SV (2001). Exploiting big biology: Integrating large-scale biological data
for function inference. Br Bioinform.

[B32] Marwah VS, Scala G, Kinaret PAS, Serra A, Alenius H, Fortino V, Greco D (2019). eUTOPIA: solUTion for Omics data PreprocessIng and
Analysis. Source Code Biol Med.

[B33] Smith ML, Baggerly KA, Bengtsson H, Ritchie ME, Hansen KD (2013). illuminaio: An open source IDAT parsing tool for Illumina
microarrays. F1000Res.

[B34] Smyth GK, Gentleman R, Carey V, Huber W, Irizarry R, Dudoit S (2005). Bioinformatics and computational biology solutions using R and
Bioconductor.

[B35] Son M-Y, Sim H, Son YS, Jung KB, Lee M-O, Oh J-H, Chung S-K, Jung C-R, Kim J (2017). Distinctive genomic signature of neural and intestinal organoids
from familial Parkinson’s disease patient-derived induced pluripotent stem
cells. Neuropathol Appl Neurobiol.

[B36] Vizkeleti L, Kiss T, Koroknai V, Ecsedi S, Papp O, Szasz I, Adany R, Balazs M (2017). Altered integrin expression patterns shown by microarray in human
cutaneous melanoma. Melanoma Res.

[B37] Wang L, Min L, Guo Q, Zhang J, Jiang H, Shao S, Xing J, Yin L, Liu J, Liu R (2017). Profiling microRNA from brain by microarray in a transgenic mouse
model of Alzheimer’s disease. Biomed Res Int.

[B38] Wang Y, Skibbe JR, Hu C, Dong L, Ferchen K, Su R, Li C, Huang H, Weng H, Huang H (2017). ALOX5 exhibits anti-tumor and drug-sensitizing effects in
MLL-rearranged leukemia. Sci Rep.

[B39] Williams M (2002). Microsoft Visual C# (Core Reference).

[B40] Yang Z, Li H, Wang Z, Yang Y, Niu J, Liu Y, Sun Z, Yin C (2018). Microarray expression profile of long non-coding RNAs in human
lung adenocarcinoma. Thorac Cancer.

[B41] Yin Z, Lan H, Tan G, Lu M, Vasilakos AV, Liu W (2017). Computing platforms for big biological data analytics:
perspectives and challenges. Comput Struct Biotechnol J.

[B42] Zhang Z, Fang C, Wang Y, Zhang J, Yu J, Zhang Y, Wang X, Zhong J (2018). COL1A1: A potential therapeutic target for colorectal cancer
expressing wild-type or mutant KRAS. Int J Oncol.

[B43] Zou D, Ma L, Yu J, Zhang Z (2015). Biological databases for human research. Genomics Proteomics Bioinform.

