# Supplementary material for: Gene Expression Analysis Platform (GEAP): A highly customizable, fast, versatile and ready-to-use microarray analysis platform
Source: Genet Mol Biol. 2021 Dec 17;45(1):e20210077. doi: 10.1590/1678-4685-GMB-2021-0077 (PMC8754388; doi:10.1590/1678-4685-GMB-2021-0077)
Supplement: Figure S1 - [file 1415-4757-GMB-45-1-e20210077-s1.pdf]

Genetics and Molecular Biology

https://doi.org/10.1590/1678-4685-GMB-2021-0077

Supplementary Material to "Gene Expression Analysis Platform (GEAP): A highly customizable, fast, versatile and ready-to-use microarray analysis platform"

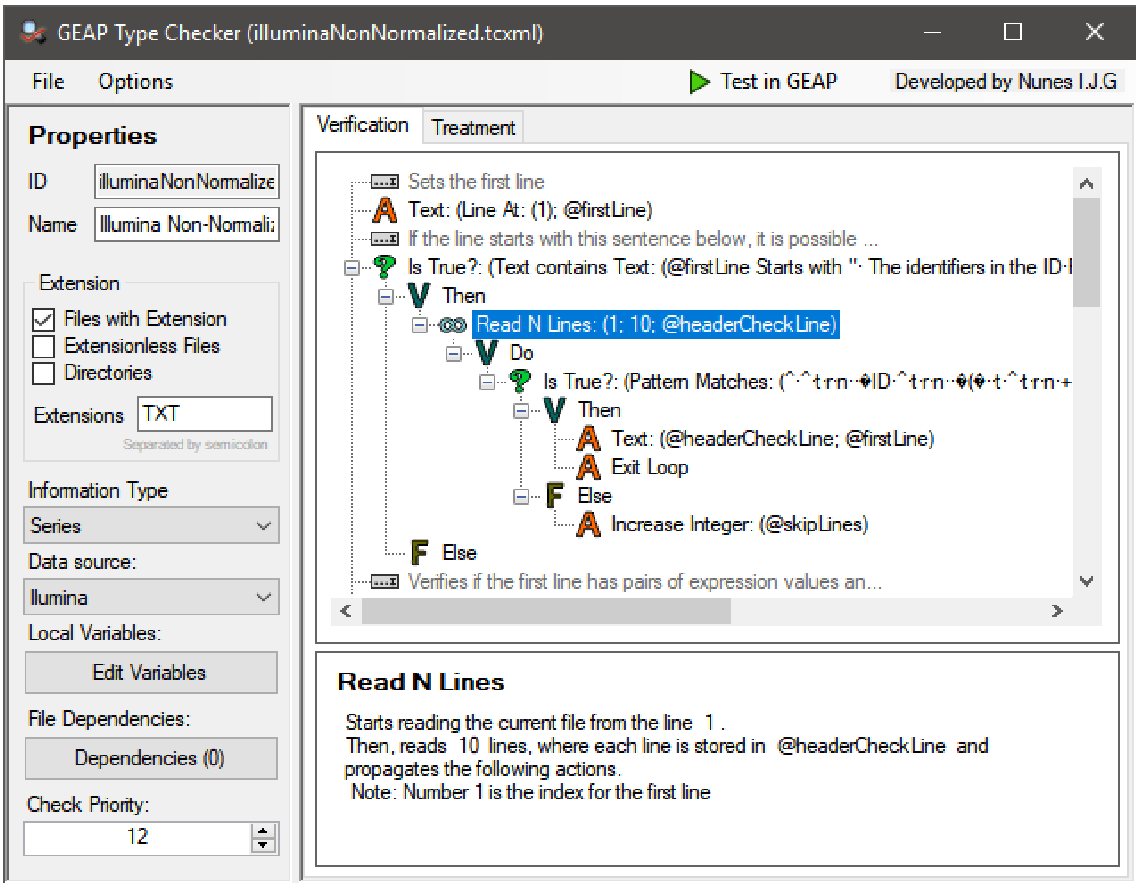

Figure S1 - TypeChecker's main workspace with an use case example. This application was developed to expand GEAP's capabilities to support multiple data sources by allowing users to create methods that check file formats. This example illustrates a set of instructions intended to validate non-normalized matrices from Illumina microarrays.
