# Supplementary material for: Gene Expression Analysis Platform (GEAP): A highly customizable, fast, versatile and ready-to-use microarray analysis platform
Source: Genet Mol Biol. 2021 Dec 17;45(1):e20210077. doi: 10.1590/1678-4685-GMB-2021-0077 (PMC8754388; doi:10.1590/1678-4685-GMB-2021-0077)
Supplement: Figure S2 - [file 1415-4757-GMB-45-1-e20210077-s2.pdf]

Genetics and Molecular Biology

https://doi.org/10.1590/1678-4685-GMB-2021-0077

Supplementary Material to "Gene Expression Analysis Platform (GEAP): A highly customizable, fast, versatile and ready-to-use microarray analysis platform"

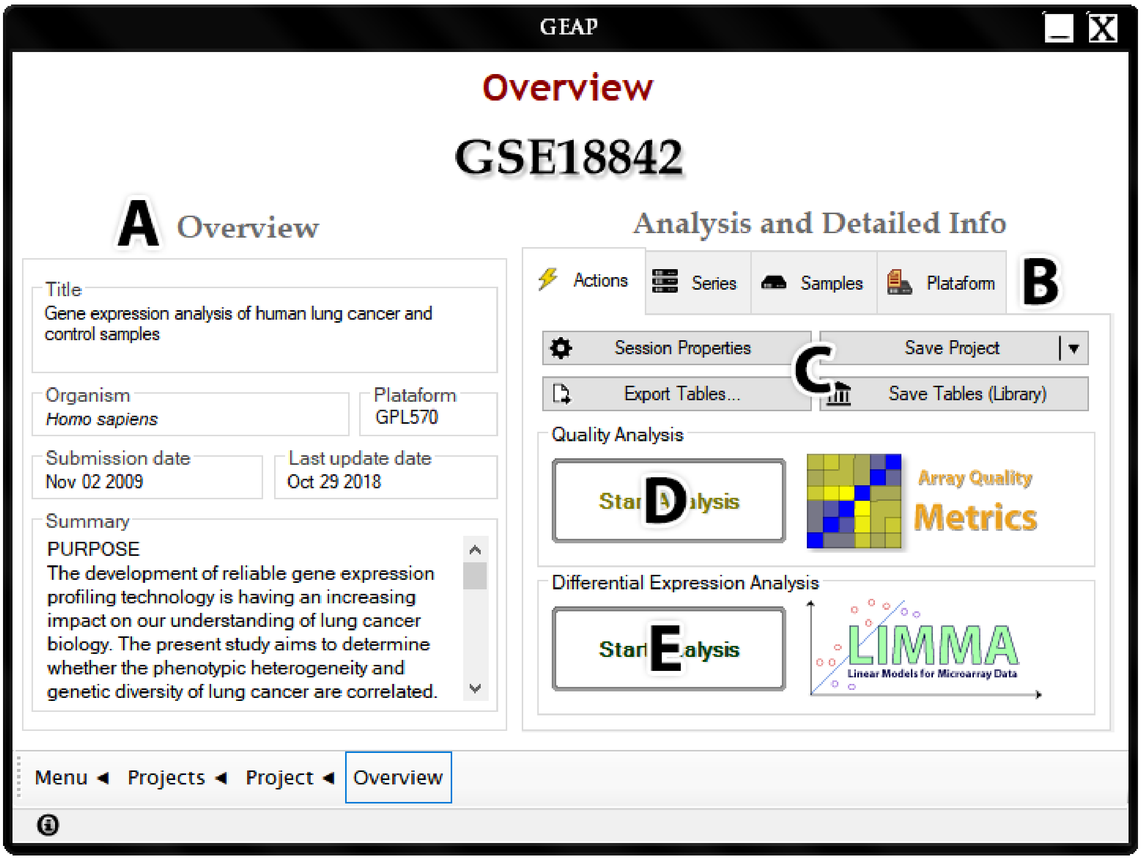

Figure S2 - Overview section that summarizes the information extracted from pre-analysis. (A) Relevant dataset metadata displayed when the study's information is present; (B) the "Actions" tab provides options for data saving and subsequent analyses, whereas the other tabs contain more specific information related to the loaded series, samples or platform; (C) session action buttons. "Session properties" provide technical information regarding the loaded session. "Save Project", "Export Tables" and "Save Tables in Library" are self-explanatory; (D) Prompts a window with options and starts the quality control analysis; and (E) redirect the user to the Differential Expression Analysis Methods section.
