# Supplementary material for: Gene Expression Analysis Platform (GEAP): A highly customizable, fast, versatile and ready-to-use microarray analysis platform
Source: Genet Mol Biol. 2021 Dec 17;45(1):e20210077. doi: 10.1590/1678-4685-GMB-2021-0077 (PMC8754388; doi:10.1590/1678-4685-GMB-2021-0077)
Supplement: Figure S3 - [file 1415-4757-GMB-45-1-e20210077-s3.pdf]

Genetics and Molecular Biology

https://doi.org/10.1590/1678-4685-GMB-2021-0077

Supplementary Material to "Gene Expression Analysis Platform (GEAP): A highly customizable, fast, versatile and ready-to-use microarray analysis platform"

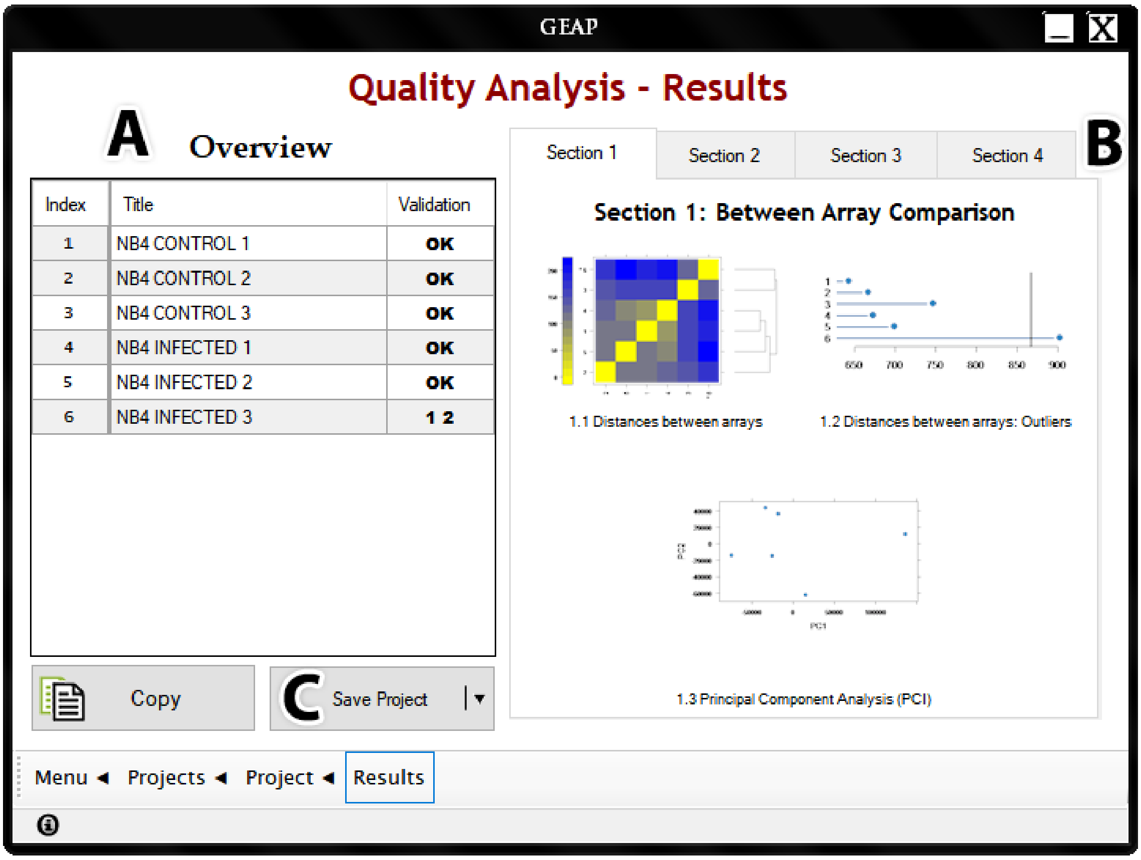

Figure S3 - Quality results section. As in the *arrayQualityMetrics* R package, the results are divided between four sections, each one validating the array quality through a specific method. (A) The results overview, where samples' quality status is listed. When outliers are present, as in the figure, they are pointed in "Validation" column; (B) the four analysis sections, equivalent to those from *arrayQualityMetrics* package; and (C) button where the results can be saved in either a new project or in an existing one.
