# Supplementary material for: Gene Expression Analysis Platform (GEAP): A highly customizable, fast, versatile and ready-to-use microarray analysis platform
Source: Genet Mol Biol. 2021 Dec 17;45(1):e20210077. doi: 10.1590/1678-4685-GMB-2021-0077 (PMC8754388; doi:10.1590/1678-4685-GMB-2021-0077)
Supplement: Figure S4 - [file 1415-4757-GMB-45-1-e20210077-s4.pdf]

Genetics and Molecular Biology

https://doi.org/10.1590/1678-4685-GMB-2021-0077

Supplementary Material to "Gene Expression Analysis Platform (GEAP): A highly customizable, fast, versatile and ready-to-use microarray analysis platform"

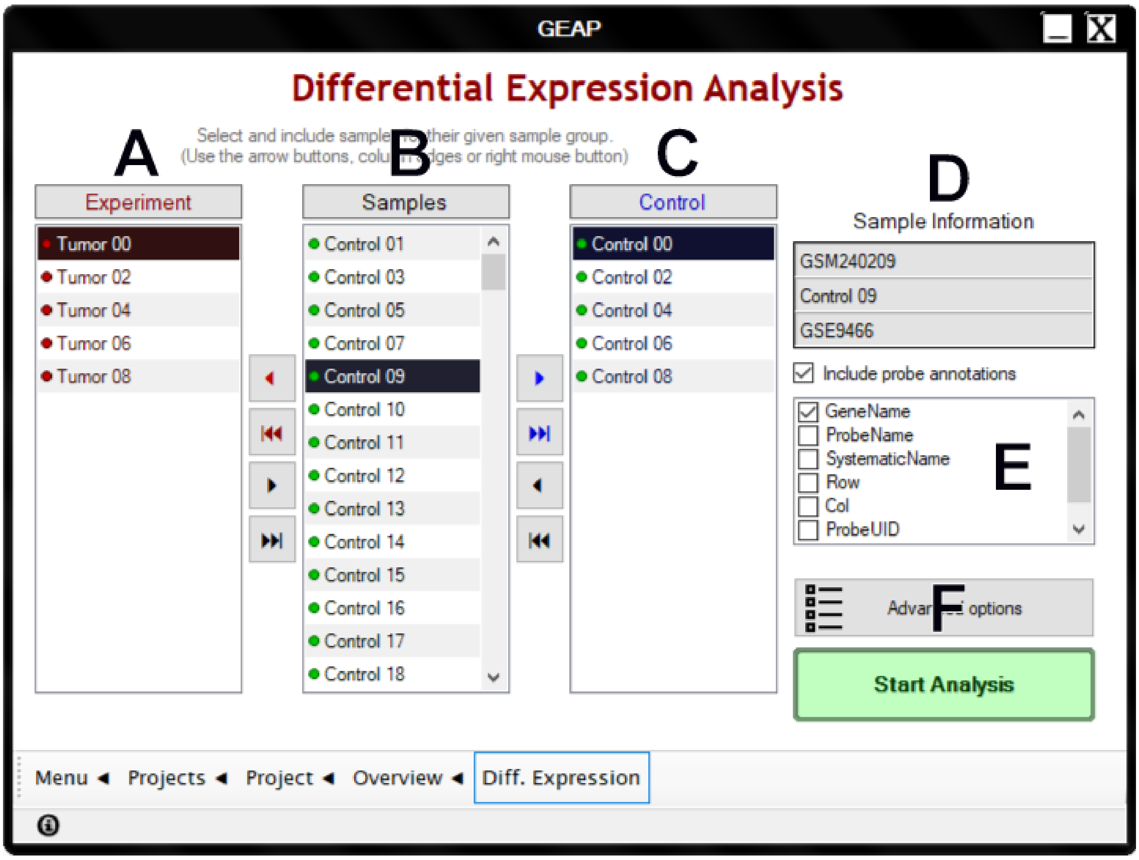

Figure S4 - Assignment of the available samples to either experimental or control group in a study case involving tumors. (A) Experimental group; (B) ungrouped samples to be distributed by the user; (C) control group; (D) list box describing the main sample information, if present; (E) probe annotations to be attached to the results, enabled when the source platform is available; and (F) advanced options, including the *p*-value correction method applied to the *p*-values.
