# Supplementary material for: Gene Expression Analysis Platform (GEAP): A highly customizable, fast, versatile and ready-to-use microarray analysis platform
Source: Genet Mol Biol. 2021 Dec 17;45(1):e20210077. doi: 10.1590/1678-4685-GMB-2021-0077 (PMC8754388; doi:10.1590/1678-4685-GMB-2021-0077)
Supplement: Figure S5 - [file 1415-4757-GMB-45-1-e20210077-s5.pdf]

Genetics and Molecular Biology

https://doi.org/10.1590/1678-4685-GMB-2021-0077

Supplementary Material to "Gene Expression Analysis Platform (GEAP): A highly customizable, fast, versatile and ready-to-use microarray analysis platform"

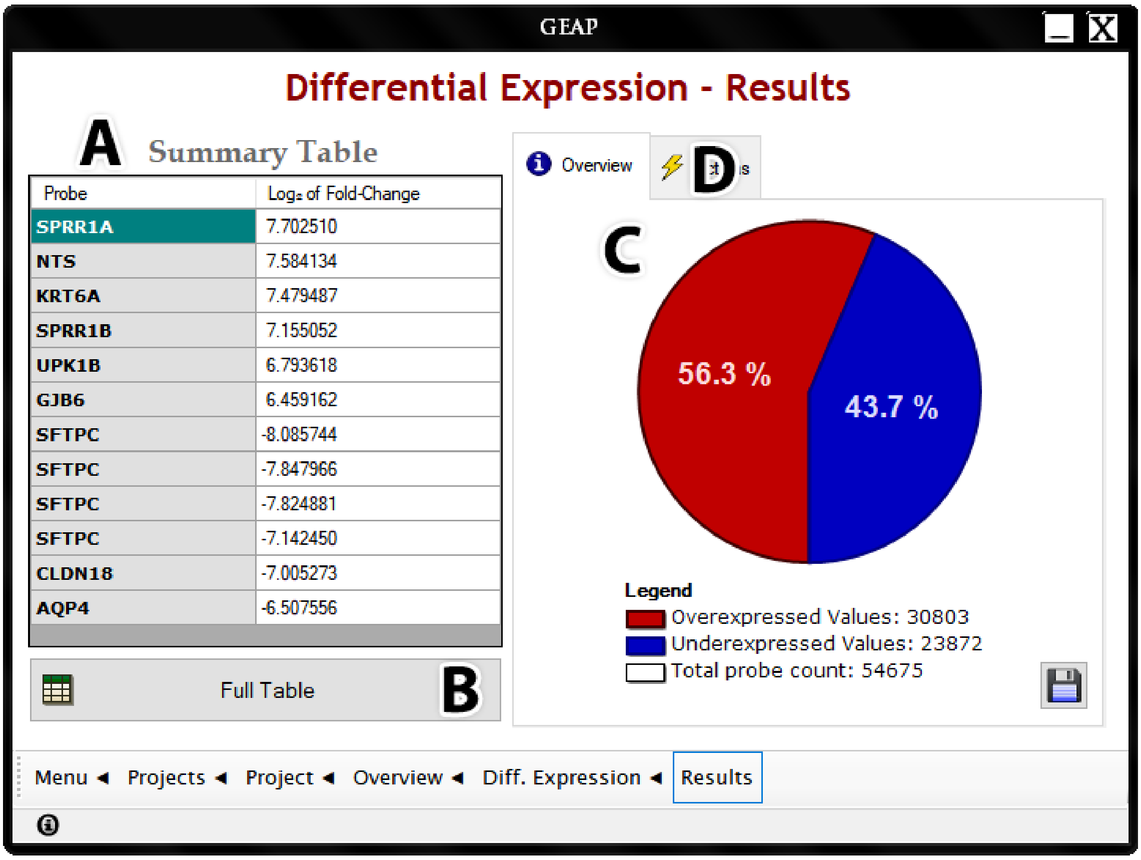

Figure S5 - General overview of the differential expression analysis results obtained by comparing the groups indicated in Figure S4. (A) Summary table, listing the probes with the highest and lowest fold-changes. Notably, if a Gene Symbol column was included as probe attribute, it is displayed instead of probe ID; (B) opens a new window to navigate over the full table (Figure 7); (C) the proportion between the fold-changes; (D) available actions to perform on the results, including saving the current results in a project.
